# Cellular senescence contributes to spontaneous repair of the rat meniscus

**DOI:** 10.1111/acel.14385

**Published:** 2024-10-22

**Authors:** Yusuke Aimono, Kentaro Endo, Ichiro Sekiya

**Affiliations:** ^1^ Center for Stem Cell and Regenerative Medicine Institute of Science Tokyo Tokyo Japan; ^2^ Center for Stem Cell and Regenerative Medicine Tokyo Medical and Dental University (TMDU) Tokyo Japan

**Keywords:** ABT‐263, cellular senescence, meniscus, senescent cell, synovial fibroblast

## Abstract

Cellular senescence, traditionally associated with aging and chronic diseases, has recently been identified as a potential facilitator of tissue regeneration via a senescence‐associated secretory phenotype (SASP). In rodents, the meniscus is known to regenerate spontaneously from the surrounding synovium, but the mechanism, and especially its relationship to cellular senescence, remains unclear. This study investigated the contribution of cellular senescence to spontaneous repair of the rat meniscus. We created a rat partial medial meniscectomy (pMx) model to evaluate time‐course changes in regenerative tissue. Immunohistochemistry revealed marked increases in p16 expression and senescence‐associated beta‐galactosidase (SA‐β‐gal) activity in the regenerating tissue at the early phase after pMx surgery. RNA sequencing of regenerating tissues identified the upregulation of genes related to aging, extracellular matrix organization, and cell proliferation. Fluorescence staining identified high expression of SOX9, a master regulator of cartilage/meniscus development, adjacent to p16‐positive cells. In vitro investigations of the effect of SASP factors on synovial fibroblasts (SFs) demonstrated that conditioned medium from senescent SFs stimulated the proliferation and chondrogenic differentiation of normal SFs. In vivo histological evaluation to determine whether selective elimination of senescent cells with a senolytic drug (ABT‐263) retarded spontaneous repair of meniscus in vivo confirmed that ABT‐263 decreased the meniscus score and expression of SOX9, aggrecan, and type 1 collagen. Our findings indicate that transient senescent cell accumulation and SASP in regenerating tissues beneficially contribute to spontaneous repair of the rat meniscus. Further research into the molecular mechanism will provide a novel strategy for meniscus regeneration based on cellular senescence.

## Graphical abstract

This study reveals that senescent synovial fibroblasts (SFs) transiently accumulate in regenerating tissue after meniscectomy in rats. Senescence‐associated secretory phenotype (SASP) factors from senescent SFs promote the proliferation and chondrogenesis of normal SFs, while the selective elimination of senescent SFs retards meniscus repair. These results indicate that transient senescent SF accumulation and SASP beneficially contribute to the spontaneous repair of the rat meniscus.

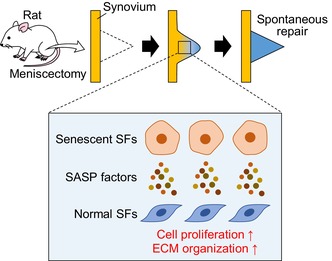

**Abbreviations**
CM: conditioned medium
COL1: type I collagen
COL2: type II collagen
DEG: differentially expressed gene
EDTA: ethylenediaminetetraacetic acid
GAG: glycosaminoglycan
GO: gene ontology
IL/TNF: IL‐1β and TNF‐α
OARSI: Osteoarthritis Research Society International
PBS: phosphate‐buffered saline
pMx: partial medial meniscectomy
qPCR: quantitative polymerase chain reaction
SASP: senescence‐associated secretory phenotype
SA‐β‐gal: senescence‐associated beta‐galactosidase
SF: synovial fibroblast
TBS‐T: Tris‐buffered saline containing 0.1% Tween‐20

## 1 INTRODUCTION

Cellular senescence is a permanent state of cell cycle arrest associated with phenotypic changes that contribute to aging and age‐related diseases. It can be triggered by various stresses, such as inflammatory stress (Paramos‐de‐Carvalho et al., 2021), oxidative stress (Chen et al., 1995), chemotherapy (Schmitt et al., 2002), and irradiation (Le et al., 2010). The most relevant feature of senescence is the development of a complex secretory program termed the senescence‐associated secretory phenotype (SASP) (Krtolica et al., 2001). Senescent cells that acquire SASP secrete a variety of inflammatory cytokines, chemokines, growth factors, and proteases (Li et al., 2023). Senescent cells that accumulate in the body interact with their surrounding microenvironment via SASP factors to participate in diverse biological phenomena. Although no clear definition presently exists to distinguish senescent from nonsenescent cells (Hernandez‐Segura et al., 2018), the proteins p16, p21, and p53 are generally used as markers of cellular senescence (Muñoz‐Espín & Serrano, 2014). In particular, p16, a cyclin‐dependent kinase 4/6 inhibitor, is one of the most reliable markers (Safwan‐Zaiter et al., 2022). Another common reliable biomarker is senescence‐associated beta‐galactosidase (SA‐β‐gal), as its activity reflects enhanced lysosomal contents (Valieva et al., 2022).

Cellular senescence has a dual nature that has attracted much attention in recent years (Paramos‐de‐Carvalho et al., 2021), with researchers in recent decades focusing in particular on the negative aspects of cellular senescence. Previous studies have demonstrated the fundamental roles of cellular senescence in various age‐related diseases, such as osteoarthritis (Coryell et al., 2021). Pathological accumulation of senescent cells is known to cause chronic inflammation and tissue degeneration via long‐lasting SASP secretion and to eventually lead to the loss of optimal tissue function. This senescent cell accumulation occurs in part due to the compromised immune system associated with aging (Vicente et al., 2016). However, recent studies have uncovered another aspect of cellular senescence that is actually beneficial for morphogenesis and tissue regeneration (Serrano, 2014). For example, the importance of cellular senescence has been demonstrated in the processes of zebrafish fin regeneration (Da Silva‐Álvarez et al., 2020) and skin wound healing in mice (Demaria et al., 2014).

These studies have confirmed the transient appearance of senescent cells in regenerating tissues and that the depletion of senescent cells impairs regeneration. The timing of the exposure to SASP is believed to be the key to these contradictory effects. The transient accumulation of senescent cells and a SASP burst can activate surrounding cells to accelerate regenerative processes (Da Silva‐Álvarez et al., 2020; Demaria et al., 2014). Recent limb regeneration studies on *Hydractinia symbiolongicarpus* and axolotl salamanders have demonstrated that SASP signals from senescent cells induce reprogramming and proliferation of the surrounding cells following amputation and drive tissue regeneration (Salinas‐Saavedra et al., 2023; Yu et al., 2023). In a more detailed study of the underlying mechanism, cardiac fibroblasts undergoing senescence by Egr1 were found to release SASP factors to regulate matrix remodeling and prevent further senescence, thereby facilitating cardiac repair (Zhang et al., 2024). Therefore, controlling these beneficial aspects of cellular senescence may emerge as a novel strategy for regenerative therapy to heal a variety of injuries, including those occurring to the meniscus of the knee.

The meniscus, a fibrocartilaginous tissue in the knee joint, plays an important role in shock absorption and joint stability. Damage or tearing of the inner two‐thirds of the meniscus does not heal spontaneously due to the avascular nature of the meniscus. The result is a mechanical overload of the articular cartilage and the subsequent development of knee osteoarthritis (Bansal et al., 2021; Stärke et al., 2009). Therefore, novel treatments that can promote meniscus regeneration are eagerly awaited. In recent years, the self‐healing potential of the meniscus in animals other than humans has gained much attention. In rodents, the gradual regeneration of a resected meniscus from the surrounding synovium has been reported (Hiyama et al., 2017), while Kim et al. reported that killing synovial fibroblasts (SFs) using a freeze–thaw method inhibited this meniscus self‐healing in rabbits (Kim et al., 2020). These studies, taken together with the high plasticity and chondrogenic potential of SFs (Noda et al., 2022), strongly suggest that SFs substantially contribute to meniscus self‐healing. Further understanding of meniscus self‐healing processes is therefore expected to lead to the development of innovative therapies for healing human meniscus injuries. However, the mechanism underlying self‐healing, and especially its relationship to cellular senescence, remains unclear.

The purpose of the present study was to elucidate the role and importance of cellular senescence in the self‐healing of the rat meniscus. We created a rat partial medial meniscectomy (pMx) model to evaluate cellular senescence in the regenerating tissues over time. We also examined the effects of SASP factors on normal SFs in vitro. In addition, we explored whether the selective elimination of senescent cells would inhibit meniscus regeneration in our rat pMx model.

## 2 METHODS

### 2.1 Animal surgery

A total of 53 wild‐type male Lewis rats (Sankyo Labo Service Corporation, Tokyo, Japan) 10–12 weeks of age were used for the experiments. All animal care and experiments were conducted in accordance with the institutional guidelines of Tokyo Medical and Dental University Animal Committee (permit no. A2022‐166C) and ARRIVE guidelines. A partial medial meniscectomy (pMx) was performed on both knees with the animal under 2% isoflurane anesthesia. For pMx surgery, the meniscotibial ligament was transected and the anterior half of the medial meniscus was removed. The joint capsule and skin were closed with 5–0 nylon sutures. At 1, 2, 4, 8, and 12 weeks after the surgery, the rats were euthanized using CO_2_. For sham surgery, the joint capsule was opened and closed. Intact rats that received no surgery served as healthy controls. All rats were allowed to walk freely in a cage. Throughout the study, all rats were housed in approved animal‐care facilities, with two rats per cage (TP‐105, TOYO‐LABO, Tokyo, Japan; measuring 280 × 440 × 205 mm [L × W × H]). The rats were provided with ad libitum access to water and a certified rat diet (Rodent Diet CE‐2, CLEA Japan, Tokyo, Japan), and were allowed to move freely within their cages.

### 2.2 Safranin O staining

Rat knee joints were fixed for 7 days at room temperature in a neutral buffer solution containing 10% formalin (Wako, Tokyo, Japan), followed by decalcification in 20% ethylenediaminetetraacetic acid (EDTA) for 21 days, and embedding in paraffin wax. The specimens were sliced into 5 μm sagittal sections and stained with safranin O (Chroma Gesellschaft Schmid & Co., Munster, Germany) and fast green (Wako). Histological scoring of the regenerating meniscus tissue was conducted using the Modified Pauli score (Hiyama et al., 2017) (Table S1). The Osteoarthritis Research Society International (OARSI) score was used to assess cartilage degeneration (Pritzker et al., 2006).

### 2.3 Immunohistochemistry

For p16, CD68, SOX9, and ACAN immunostaining, antigen retrieval was performed by immersing sections in a solution of 10 mM Tris plus 1 mM EDTA (pH 9.0) and heated to 60°C for 16 h. For type I and II collagen (COL1 and COL2), the sections were treated with proteinase K (Dako) for 10 min at room temperature. For type II collagen, the sections were further incubated with 5 mg/mL hyaluronidase (Sigma‐Aldrich, Saint Louis, MO, USA) for 2 h at room temperature. The slides were immersed in methanol containing 0.3% H_2_O_2_ for 30 min and then washed with Tris‐buffered saline containing 0.1% Tween‐20 (TBS‐T). After blocking with Blocking One Histo (NACALAI TESQUE, Kyoto, Japan), the sections were incubated overnight at 4°C with primary antibody against p16 (1:100; Abcam, Cambridge, UK), CD68 (1:200; Abcam), SOX9 (1:500; Chemicon, Billerica, MA, USA), ACAN (1:400; Proteintech, Rosemont, IL, USA), type I collagen (1:200; Rockland, Philadelphia, PA, USA), or type II collagen (1:50; F‐57; Kyowa Pharma Chemical, Toyama, Japan). After three washes with TBS‐T, the sections were incubated with secondary antibodies conjugated with horseradish peroxidase (1:200; Abcam) for 1 h at room temperature. The sections were stained with diaminobenzidine (DAB) solution (Dako) for 5 min and then counterstained with hematoxylin. For fluorescence staining, the sections were incubated at 4°C overnight with primary antibody against p16 (1:100; Abcam) and SOX9 (1:500; Chemicon). After three washes with TBS‐T, the sections were incubated at room temperature for 1 h with secondary antibodies conjugated with Alexa488 (1:200; Abcam) and Alexa555 (1:200; Abcam) and fixed with Vectashield (Vector Laboratories) mounting medium. All images were captured using a BZ‐X700 microscope (Keyence, Osaka, Japan) and analyzed using Fiji/ImageJ. DAB‐positive areas were quantified in three fields of view (100 × 100 μm or 150 × 150 μm) for p16, CD68, ACAN, COL1, and COL2. The percentage of SOX9‐positive cells was quantified in three fields of view (100 × 100 μm). We quantified the SOX9‐positive rate by randomly selecting ten p16‐positive (senescent) and ten p16‐negative (non‐senescent) cells in each section and counting the total number of cells and the SOX9‐positive cells within a radius of 20 μm of the selected cells. The counts per section from the selected areas were summed to calculate the SOX9‐positive rate.

### 2.4 Senescence‐associated β‐galactosidase staining

SA‐β‐gal staining was performed using a Senescence β‐Galactosidase Staining Kit (Cell Signaling Technology, Danvers, MA, USA). Tissues were fixed with fixative solution for 30 min and incubated in pH 6.0 staining solution at 37°C for 4 h. Immediately after staining, the tissues were photographed using a stereomicroscope (M165FC; Leica, Wetzlar, Germany). The tissues were then formalin‐fixed, sectioned, and stained with Kernechtrot Stain Solution (Muto Pure Chemicals, Tokyo, Japan) for 5 min to visualize the cell nuclei.

### 2.5 RNA sequencing

Regenerating tissues were harvested from knees 1 week after pMx surgery, as was synovium around the medial meniscus from intact knees and from knees 1 week after sham surgery. Because the boundary between the regenerated tissue and the posterior horn of the meniscus was grossly visible, the regenerating tissue was excised at the boundary and the surrounding synovium was carefully removed. The tissues were homogenized with a Power Masher II (Nippi, Tokyo, Japan), and total RNA was extracted using a miRNeasy Mini Kit (Qiagen) according to the manufacturer's instructions. The quality of the extracted RNA is shown in Table S2. The RNA sequencing analysis was conducted by the Beijing Genomics Institute (BGI; Shenzhen, Guangdong, China). The mRNA was captured using oligo‐dT beads, fragmented, and used for the synthesis of single‐strand cDNA using random N6‐primed reverse transcription, followed by a second‐strand cDNA with dUTP. After PCR amplification, the resulting products underwent sequencing on the DNBSEQ platform. Data were analyzed utilizing Dr. Tom, a BGI data‐mining system. The gene expression levels were calculated as transcripts per million values. Differentially expressed genes (DEGs) were determined between each group (|Log2FC| >1, *Q* value <0.05). Gene Ontology (GO) analysis was performed on the DEGs of pMx versus sham that did not overlap with the DEGs of sham versus intact. Senescence‐associated genes were identified using SenMayo (Saul et al., 2022) and the genes coding secreted factors were extracted using the Matrisome database (Shao et al., 2020).

### 2.6 Isolation of rat synovial fibroblasts

Rat synovium was digested with 3 mg/mL *Clostridium histolyticum*‐derived collagenase type V (Sigma‐Aldrich, Saint Louis, MO, USA) at 37°C for 3 h. Debris was then removed by passing the digest through a 70 μm cell strainer (Greiner Bio‐One, Kremsmünster, Austria), and the filtrate containing the cells was collected. The cells were washed with phosphate‐buffered saline (PBS) and plated at a density of 2000 cells/cm^2^ in 145 cm^2^ dishes in a growth medium consisting of α‐modified essential medium (Thermo Fisher Scientific, Waltham, MA, USA), 10% fetal bovine serum (Thermo Fisher Scientific), and 1% antibiotic–antimycotic (Thermo Fisher Scientific). The cells were cultured until confluent and then detached with 0.25% trypsin and 1 mM EDTA (Thermo Fisher Scientific). Cells at passage 1–2 were used for the experiments.

### 2.7 Senescence induction and conditioned medium preparation

Normal rat SFs were induced to senesce by culturing for 5 days in growth medium with 1 ng/mL recombinant rat IL‐1β and TNF‐α (IL/TNF; Peprotech, Rocky Hill, NJ, USA). After two washes with PBS, the cells were cultured in growth medium without IL/TNF for 24 h. The culture medium (CM) was collected as IL/TNF‐CM and stored at −30°C. Cont‐CM was collected in the same manner from normal SF cultures.

### 2.8 Cell proliferation assay

A Cell Counting Kit‐8 (Dojindo, Tokyo, Japan) was used to assess cell proliferation. Rat SFs were seeded into 96‐well plates at a density of 500 cells/cm^2^ and cultured for 24 h. After incubation for 2, 4, and 6 days, the absorbance at 450 nm wavelength was measured using a plate reader (Infinite M200; Tecan, Männedorf, Switzerland). The cells were then formalin fixed and stained with crystal violet.

### 2.9 Chondrogenic differentiation assay

Rat SFs were seeded at 1 × 10^5^ cells/well in 96‐well PrimeSurface (Sumitomo Bakelite, Tokyo, Japan) and cultured in a chondrogenic induction medium consisting of high‐glucose Dulbecco's Modified Eagle Medium (Thermo Fisher Scientific), 1% insulin–transferrin–selenium (BD Biosciences), 50 μg/mL ascorbate‐2‐phosphate, 40 μg/mL L‐proline (Sigma‐Aldrich), 100 nM dexamethasone, 100 μg/mL pyruvate (Sigma‐Aldrich), 1% antibiotic–antimycotic, 10 ng/mL transforming growth factor‐β3 (Miltenyi Biotec, Bergisch Gladbach, Germany), and 500 ng/mL bone morphogenetic protein‐2 (Medtronic, Minneapolis, MN, USA). After 2 weeks of cultivation, the formed spheroids were subjected to safranin O/fast green staining, glycosaminoglycan (GAG) and DNA quantification, and quantitative polymerase chain reaction (qPCR).

### 2.10 Biochemical analysis

Spheroids were digested with 100 μg/mL papain (Sigma‐Aldrich) at 65°C for 16 h. Subsequently, fluorescence intensity was assessed using a microplate reader (Tecan, Männedorf, Switzerland) at an excitation wavelength of 360 nm and an emission wavelength of 465 nm. A standard curve was generated using calf thymus DNA (Sigma‐Aldrich). The GAG content was then measured using a Blyscan Kit (Biocolor, Westbury, NY, USA) according to the manufacturer's instructions. The optical density was assessed at 656 nm with a microplate reader. To compare the GAG‐producing ability, the GAG content was standardized relative to the DNA content (GAG/DNA).

### 2.11 qPCR

Total RNA was extracted using the miRNeasy Mini Kit according to the manufacturer's instructions. First‐strand complementary DNA was synthesized from total RNA using a ReverTra Ace qPCR RT Master Mix with gDNA Remover (Toyobo, Osaka, Japan). The qPCR was performed with THUNDERBIRD SYBR qPCR Mix (QPS‐201; Toyobo) on a Lightcyler 480 System II (Roche Diagnostics). The following primers were used in this study: SOX9, 5′‐ATCTTCAAGGCGCTGCAA‐3′ (forward) and 5′‐CGGTGGACCCTGAGATTG‐3′ (reverse); ACAN, 5′‐TGGCTGCAGGACCAGACT‐3′ (forward) and 5′‐CGCCATAGGTCCTGACTCC‐3′ (reverse); COL2A1, 5′‐CTTTCCTCCGTCTACTGTCCA‐3′ (forward) and 5′‐GCCCTCATCTCCACATCATT‐3′ (reverse); COL1A1, 5′‐TCCTGCCGATGTCGCTATC‐3′ (forward) and 5′‐CCATGTAGGCTACGCTGTTCTTG‐3′ (reverse); COL10A1, 5′‐GGTCCACCAGGTCCACAA‐3′ (forward) and 5′‐TGGCTTCCCAATACCTTCTC‐3′ (reverse); β‐actin, 5′‐GCAGGAGTACGATGAGTCCG‐3′ (forward) and 5′‐ACGCAGCTCAGTAACAGTCC‐3′ (reverse); p16, 5′‐GCTGGATGTGCGCGATGCC‐3′ (forward) and 5′‐CAGAAGTTATGCCTGTCGGTGACC‐3′ (reverse); p21, 5′‐TCCTGGTGATGTCCGACCTGTTC‐3′ (forward) and 5′‐GCGGCTCAACTGCTCACTGTC‐3′ (reverse); p53, 5′‐AGAGAGCACTGCCCACCA‐3′ (forward) and 5′‐AACATCTCGAAGCGCTCAC‐3′ (reverse); GLB1, 5′‐CGCTGCGCACAGTTTCTATAATG‐3′ (forward) and 5′‐ATGCTTCCCGAGATGTAGCG‐3′ (reverse); 18S ribosomal RNA, 5′‐CACGGGTGACGGGGAATCAG‐3′ (forward) and 5′‐CGGGTCGGGAGTGGGTAATTTG‐3′ (reverse). The cycling conditions were 45 cycles at 95°C for 15 s and then 60°C for 30 s. β‐actin and 18 s ribosomal RNA were used as the endogenous control genes.

### 2.12 Intraarticular injections of ABT‐263

ABT‐263 was obtained from MedChemExpress (Monmouth Junction, NJ, USA) and dissolved in DMSO (Wako, Tokyo, Japan). Rats received intra‐articular injections of 2.5 mM ABT‐263 (50 μL) and vehicle control (5% DMSO, 40% PEG300, 5% Tween 80 [Selleck, Houston, USA], and 50% saline) twice a week from 1 week after pMx surgery. Regenerating tissues were subjected to qPCR (2 weeks postoperatively) and histological analysis (8 and 12 weeks postoperatively).

### 2.13 Statistical analysis

All statistical analyses were conducted using GraphPad Prism 10 (GraphPad Software, CA, USA). Comparisons between two groups were made using an unpaired *t*‐test. Comparisons between multiple groups were done using one‐way ANOVA, followed by Tukey's or Dunnett's multiple comparison test. *p* values <0.05 were considered statistically significant.

## 3 RESULTS

### 3.1 Time‐course histological changes in spontaneous repair of the rat meniscus

**FIGURE 1:**
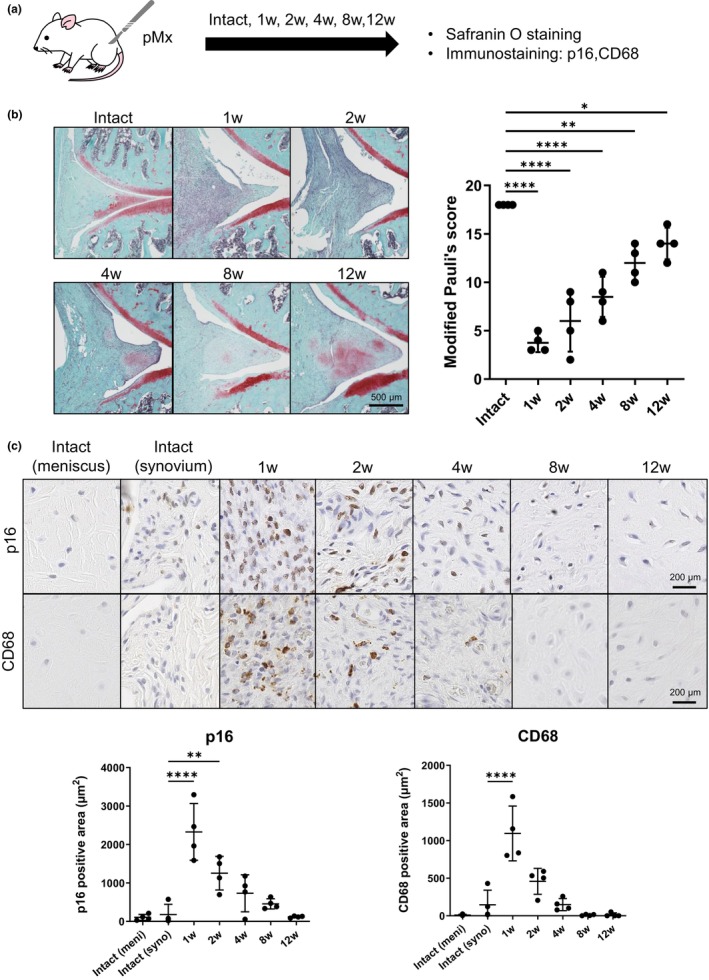
Time course of the histological changes during spontaneous repair of the rat meniscus. (a) Scheme of the experiment. A rat partial meniscectomy (pMx) model was created, and histological evaluation was performed on regenerated tissue from 1 to 12 weeks after the surgery. (b) Regenerative images of safranin O staining and the modified Pauli score. (c) Representative images of p16 and CD68 immunostaining and quantification of the positive areas. The inner part of the meniscus, the adjacent synovium, and regenerative tissues were evaluated. *p < 0.05, **p < 0.01, ****p < 0.0005.

Spontaneous repair of the meniscus was histologically evaluated over 12 weeks in the rat pMx model (Figure 1a). Safranin O staining revealed the existence of regenerative meniscal tissue from 1 week postoperatively (Figure 1b). The regenerative tissue that grew from the surrounding synovium showed higher cell density, less staining intensity, and a more irregular surface compared to the intact meniscus. The cellularity and surface irregularity decreased over the 12 week study interval. Chondrocyte‐like cells that positively stained for GAG with safranin O were observed from 4 weeks postoperatively. The staining increased at 8 and 12 weeks. The modified Pauli score significantly decreased at 1 week postoperatively compared with the intact group and improved over time for the 12 weeks. The areas showing p16 and CD68 expression in regenerating tissues and intact meniscus/synovium, used to assess the number of senescent cells and macrophages (Figure 1c), increased markedly at 1 week postoperatively and then decreased gradually. Quantification of the positive areas showed that both p16 and CD68 staining at 1 week postoperatively was significantly greater in regenerated tissue than in the intact synovium.

### 3.2 Early histological changes in intact, sham, and pMx knees

**FIGURE 2:**
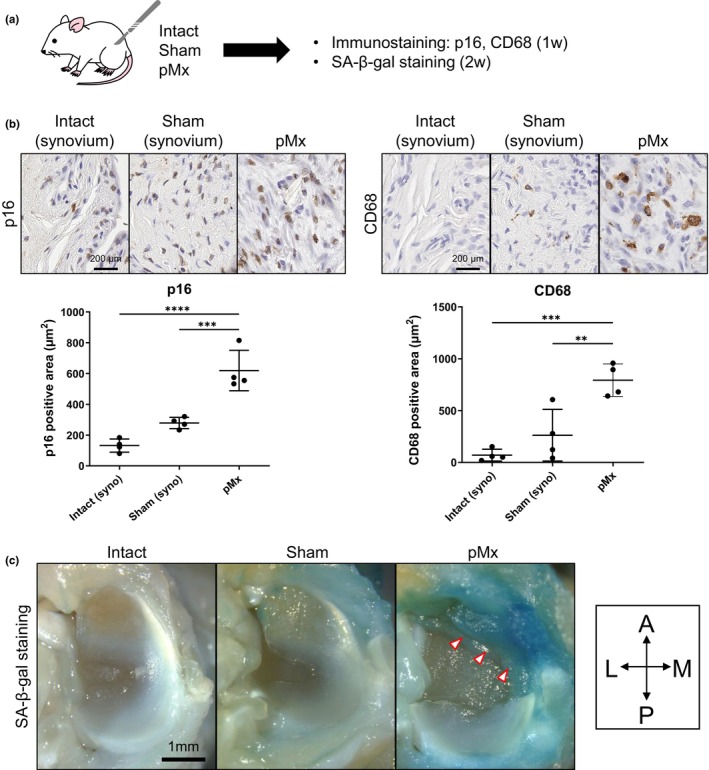
Early histological changes of intact, sham, and partial meniscectomy (pMx) knees. (a) Scheme of the experiment. Immunostaining and SA‐β‐gal staining were performed at 1 and 2 weeks postoperatively. (b) Representative images of p16 and CD68 immunostaining and quantification of the positive areas. The synovium adjacent to the meniscus and regenerative tissues were evaluated. (c) Representative images of senescence‐associated beta‐galactosidase (SA‐β‐gal) staining. *p < 0.05, **p < 0.01, ***p < 0.005, *****p < 0.0005. A, anterior; P, posterior; M, medial; L, lateral.

The effects of surgical intervention on cellular senescence were investigated by immunostaining and SA‐β‐gal staining of intact knees and knees at 1 week after sham or pMx surgery (Figure 2a). For both p16 and CD68, the staining area tended to be larger for the sham synovium than for the intact synovium (Figure 2b). However, the regenerative tissue in the pMx group showed a much larger staining area compared with either the intact or the sham synovium, and the difference was statistically significant. Double staining of the regenerated tissue for p16 and CD68 showed a small overlap of both signals (Figure S1). Almost no SA‐β‐gal staining was observed for the intact meniscus and synovium (Figure 2c). The sham group showed mild staining in the synovial tissue adjacent to the medial meniscus, whereas the pMx group showed strong staining in the regenerating tissues. Histological observation of the SA‐β‐gal‐stained knees showed similar results; however, the staining was limited to the superficial layer (Figure S2).

### 3.3 Comprehensive gene expression analysis of regenerative tissue

**FIGURE 3:**
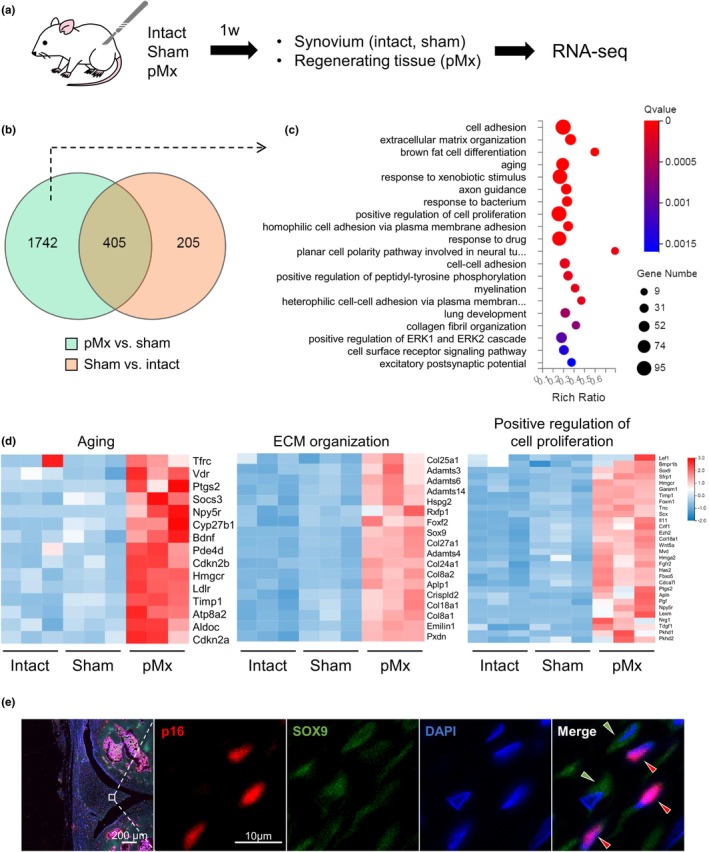
Transcriptome analysis of regenerative tissue. (a) Scheme of the experiment. RNA sequencing was performed on intact knees and knees at 1 week after sham and partial meniscectomy (pMx) surgery. (b) Venn diagram of differentially expressed genes (DEGs) between the intact, sham, and pMx groups. (c) Gene ontology analysis of 1742 genes specifically upregulated in the pMx group. The top 20 terms are shown as a bubble plot. (d) Heatmaps showing DEGs associated with “aging,” “ECM organization,” and “cell proliferation.” (e) Representative images of p16 and SOX9 fluorescence staining.

We performed RNA sequence analysis for intact knees and knees 1 week after sham and pMx surgery to analyze the genes that were specifically expressed in regenerative tissue (Figure 3a). The number of DEGs was 607 for sham versus intact and 2147 for pMx versus sham, with 405 DEGs in common (Figure 3b). We eliminated the effect of surgical intervention by excluding 405 DEGs common to sham versus intact from the 2147 DEGs in pMx versus sham, resulting in a total of 1742 genes for further analyses. GO analysis revealed an enrichment of genes related to extracellular matrix organization, aging, and positive regulation of cell proliferation (Figure 3c). Among the genes associated with aging, several, including Cdkn2a (p16), were specifically upregulated in the pMx group (Figure 3d). Many collagen‐coding genes involved in extracellular matrix organization, as well as the transcription factor Sox9, were highly expressed in the pMx group. A number of genes related to cell proliferation, including SOX9, also showed upregulated expression in the pMx group. In addition, SenMayo identified 22 DEGs that overlapped in a senescence/SASP gene set, six of which were upregulated in the pMx group (Figure S3). At the same time, various genes coding secreted factors were specifically upregulated in the pMx group (Figure S4). These results prompted us to immunostain the regenerating tissues of the pMX group for p16 and SOX9 (Figure 3e). The regenerating tissues showed the presence of p16‐positive cells surrounded by cells strongly expressing SOX9. We quantified this by calculating the SOX9 positivity of cells surrounding p16‐positive (senescent) cells and p16‐negative (non‐senescent) cells. The SOX9‐positive rate was higher for cells surrounding p16‐positive cells than for cells surrounding p16‐negative cells (Figure S5).

### 3.4 Effect of senescence‐associated secretory phenotype factors derived from senescent synovial fibroblasts

**FIGURE 4:**
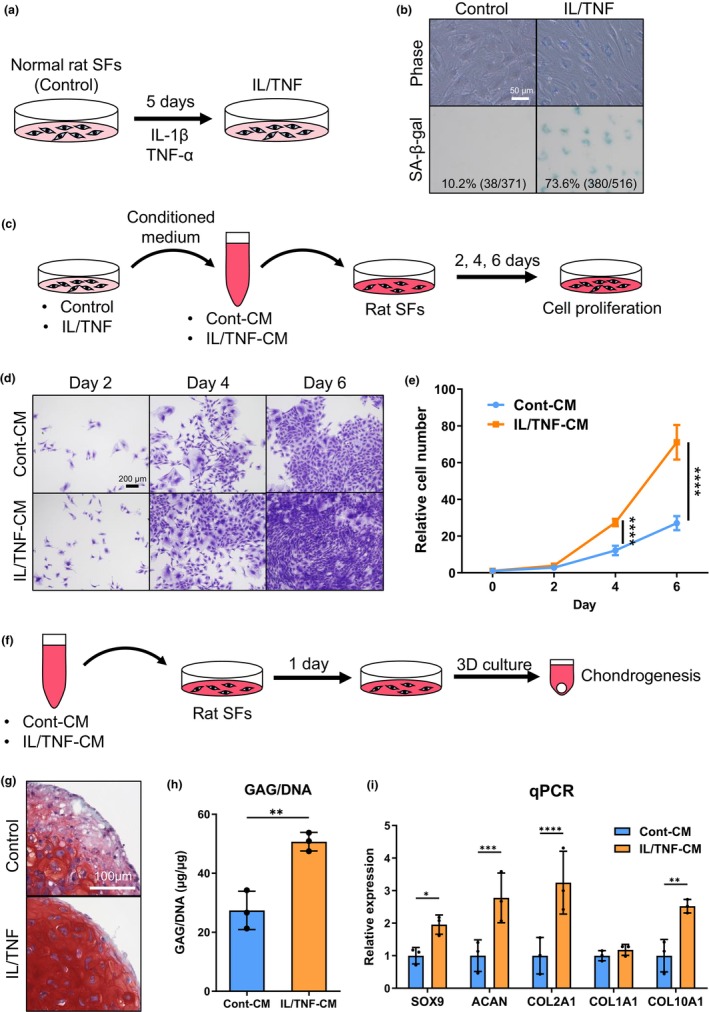
Effect of senescence‐associated secretory phenotype (SASP) factors derived from senescent rat synovial fibroblasts (SFs). (a) Schema of senescence induction in rat SFs using IL‐1β and TNF‐α (IL/TNF). (b) Phase contrast and brightfield images of senescence‐associated beta‐galactosidase (SA‐β‐gal) staining. The percentage of SA‐β‐gal–positive cells is shown. (c) Scheme of the cell proliferation assay. Rat SFs were cultured in conditioned medium collected from the control and IL/TNF SFs (cont‐CM and IL/TNF‐CM) for 2, 4, and 6 days. (d) Representative images of cells stained with crystal violet. (e) Relative cell numbers at days 0, 2, 4, and 6. (f) Scheme of chondrogenic differentiation assay. Rat SFs were pretreated with cont‐CM and IL/TNF‐CM for 1 day and then subjected to 3D chondrogenic culture. (g) Representative images of safranin O staining of chondrogenic spheroids. (h) Quantification of the glycosaminoglycan (GAG)/DNA ratio. (i) Relative gene expression of SOX9, ACAN, COL2A1, COL1A1, and COL10A1. *p < 0.05, **p < 0.01, ***p < 0.005, ****p < 0.0005. Each experiment was performed in triplicate and repeated twice. Representative data are presented.

We also assessed the effect of SASP factors released by senescent SFs on normal SFs. We first induced senescence in rat SFs by treating them with IL/TNF (Figure 4a). After 5 days of IL/TNF treatment, the cells assumed a larger and flatter morphology, and their SA‐β‐gal staining intensity increased markedly compared to untreated control cells (Figure 4b). Further evaluation of the proliferation of normal rat SFs exposed to CM from control and IL/TNF‐treated SFs (cont‐CM and IL/TNF‐CM) for 2, 4, and 6 days (Figure 4c) revealed a more rapid proliferation of rat SFs cultured in IL/TNF‐CM than in cont‐CM (Figure 4d). The relative cell number was significantly larger in the IL/TNF‐CM group at Days 4 and 6 (Figure 4e).

The effect of CM on chondrogenic potential was also evaluated by pretreating rat SFs with cont‐CM and IL/TNF‐CM for 24 h (Figure 4f). The safranin O staining was more intense, and the GAG/DNA ratio was significantly larger for the cells pretreated with IL/TNF‐CM than with cont‐CM (Figure 4g,h). The expression of chondrogenic genes (SOX9, ACAN, COL2A1, and COL10A1) was also significantly upregulated in the IL/TNF‐CM pretreatment group (Figure 4i).

### 3.5 Effect of the senolytic drug ABT‐263 on spontaneous meniscus repair

**FIGURE 5:**
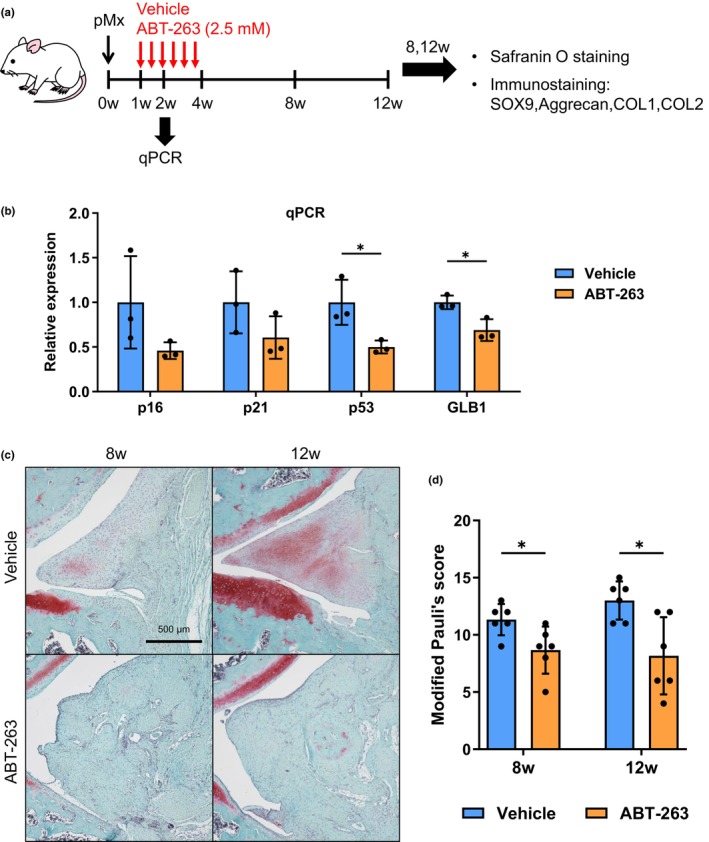
Effect of ABT‐263 on spontaneous meniscus repair. (a) Scheme of the experiment. Rats received intra‐articular injections of ABT‐263 twice a week from 1 week after partial meniscectomy (pMx) surgery. Regenerative tissues were subjected to qPCR at 2 weeks and histological analysis at 8 and 12 weeks. (b) Expression levels of senescence‐related genes (p16, p21, and p53). (c) Representative images of safranin O staining of regenerative tissues at 8 and 12 weeks. (d) Modified Pauli score at 8 and 12 weeks. *p < 0.05.

**FIGURE 6:**
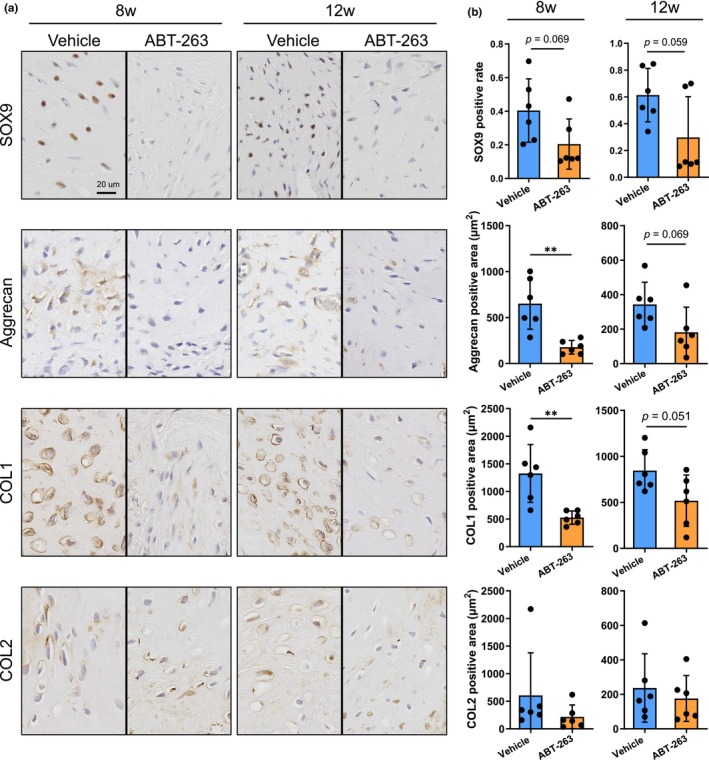
Immunostaining of regenerative tissues. (a) Representative images of SOX9, Aggrecan, COL1, and COL2 at 8 and 12 weeks. (b) Quantification of SOX9‐positive cell rate and positive areas for aggrecan, collagen type 1 (COL1), and collagen type 2 (COL2) at 8 and 12 weeks. *p < 0.05, **p < 0.01.

The significance of cellular senescence in spontaneous meniscus repair was investigated by eliminating senescent cells with ABT‐263 from 1 to 4 weeks after the pMx surgery, when the senescent cells were most abundant (Figure 5a). ABT‐263 has been reported to selectively eliminate senescent mouse and human SFs (Gil et al., 2022; Miura et al., 2022). The dose number and concentration were determined as described in a previous rat study (Yang et al., 2020). The expression levels of senescence‐related genes (p16, p21, and p53) in the regenerated tissue at 2 weeks were lower in the ABT‐263 group than in the vehicle group (Figure 5b). The ABT‐263 treatment also decreased the expression of GLB1 encoding β‐galactosidase. Safranin O staining confirmed a more uneven surface and weaker staining in the ABT‐263 group than in the vehicle group (Figure 5c). Fewer cartilage‐like cells and more fibroblast‐like cells were observed in the regenerated tissue of the ABT‐263 group at 8 and 12 weeks. Histological scores for regenerating the meniscus were significantly lower in the ABT group than in the vehicle group at both 8 and 12 weeks postoperatively (Figure 5d). The immunostaining results showed decreased expression of SOX9, ACAN, and COL1 in the ABT‐263 group (Figure 6a,b). Conversely, no obvious differences were observed in COL2 expression between the two groups. Cartilage degeneration scores showed no significant differences at 8 or 12 weeks (Figure S6).

## 4 DISCUSSION

In this study, we first evaluated the time course of the histological changes during spontaneous repair in a rat pMx model. Regenerative tissues showed deeper staining for safranin O, lower cellularity, and a smoother surface over time, which provided evidence of the self‐healing ability of the rat meniscus. Although no studies have investigated spontaneous repair of the rat meniscus, these observations were similar to the results obtained for spontaneous meniscus repair in mice (Hiyama et al., 2017). At 1 week postoperatively, the regenerative tissue grew from the surrounding synovium, suggesting that SFs are the key players in meniscus regeneration (Kim et al., 2020).

Immunohistochemistry revealed significantly increased p16‐positive areas at 1 week postoperatively in the regenerating tissue of the pMx group compared with the intact and sham groups. SA‐β‐gal staining also showed dark staining in the regenerated tissue with the surrounding synovium. These results clearly indicated that senescent cells were transiently accumulated, especially in the early stages of meniscus regeneration, suggesting a possible regulatory role for these cells in meniscus regeneration. The p16‐positive areas then gradually decreased during the remaining 11 weeks. According to a previous report, macrophages are a major contributor to senescent cell clearance (Elder & Emmerson, 2020). The CD68‐positive area exhibited the same behavior as the p16‐positive area, suggesting that the senescent cells in regenerating tissues may have been removed by macrophages. The slight overlap of the p16 and CD68 signals revealed the presence of a few p16‐positive macrophages; however, most of the p16‐positive cells were not macrophages.

We performed RNA‐seq and analyzed the DEGs specific to pMx versus sham to eliminate the effects of surgical intervention. GO analysis showed the enrichment of genes related to aging in the regenerating tissues of the pMx group. Our observation of upregulated expression of Cdkn2a (p16), together with the immunohistochemistry and SA‐β‐gal staining results, indicated that cellular senescence had almost certainly occurred in regenerative tissues early after the pMx surgery. Extracellular matrix organization‐related and cell proliferation‐related genes were also enriched in the pMx group, and Sox9 expression was common to both groups. Sox9 is an important master transcription factor for chondrocyte differentiation/proliferation and meniscus regeneration (Muhammad et al., 2014; Song & Park, 2020; Yan et al., 2023). Thus, regenerative tissues were expected to be rich in signals essential for tissue regeneration.

The fluorescence staining experiment revealed that the SOX9‐positive cells were surrounded by p16‐positive cells, suggesting that the source of these signals was the senescent cells. We examined this possibility in vitro by first inducing senescence in rat SFs using IL‐1β and TNF‐α to recapitulate the inflammatory environment after the pMx surgery (Huang et al., 2020; Lou et al., 2020). Cells in the IL/TNF group were large and displayed a flattened morphology and dark staining for SA‐β‐gal, all of which are specific features of senescent cells (Muñoz‐Espín & Serrano, 2014). Our further evaluation of the effect of SASP factors on normal rat SFs using CM from the control and IL/TNF groups revealed that IL/TNF‐CM clearly stimulated cell division, cartilaginous ECM synthesis, and chondrogenic gene expression. These data demonstrated that SASP factors released from inflammation‐induced senescent SFs were beneficial for cell proliferation and chondrogenic differentiation of normal SFs. In particular, the upregulation of Sox9 by the treatment with IL/TNF‐CM, taken together with the RNA‐seq and fluorescent staining results, suggested that senescent SFs provoke the expression of Sox9 via SASP factors both in vitro and in vivo.

The link between cellular senescence and SOX9 has been reported to influence the progression of tissue repair/degeneration. For cartilage, SOX9 downregulation drives chondrocyte senescence, thereby contributing to cartilage degeneration or preventing cartilage repair (Ashraf et al., 2016). Conversely, Faleeva et al. showed that SOX9 expression in vascular smooth muscle cells led to vascular aging due to stiffening of the ECM (Faleeva et al., 2024). The role of SOX9 in aging is not consistent and may vary depending on the cell type, microenvironment, and timing. Nevertheless, our results support the critical role of cellular senescence and SOX9 in spontaneous meniscus repair.

Finally, we investigated the importance of senescent SFs in meniscus regeneration by eliminating the SFs with ABT‐263, a senolytic drug that is reported to eliminate senescent SFs (Chang et al., 2016; Miura et al., 2022; Zhu et al., 2016). ABT‐263 was administered from 1 to 4 weeks postoperatively because the p16‐positive senescent cells increased histologically during that period. Quantitative PCR at 2 weeks confirmed decreased expression of senescence markers in the ABT‐263 group, indicating an effective elimination of senescent SFs. The regenerated meniscus was less organized, and the histological scores at both 8 and 12 weeks were significantly lower in the ABT‐263 group than in the untreated group. Consistent with this, the expression of cartilage and meniscus markers (i.e., SOX9, Aggrecan, COL1) was lower in the ABT‐263 group. These results indicated that the inhibitory effects probably arose due to the removal of senescent SFs and a resulting inadequate exposure to SASP factors. Thus, the transient accumulation of senescent cells and consequent short‐term SASP release are considered very important for spontaneous repair of the rat meniscus.

Eliminating senescent cells with ABT‐263 was detrimental in the present study; however, this elimination was reported as beneficial in a rat osteoarthritis model, thereby highlighting the dual nature of cellular senescence (Yang et al., 2020). However, in our study, cartilage degeneration scores were not improved by the ABT‐263 treatment. Two possible reasons may explain this discrepancy. One is that our duration of ABT‐263 administration was limited to 1–4 weeks postoperatively, whereas senolytic drugs are known to require continuous administration to exert and maintain their effects. The second reason is that ABT‐263 delayed meniscus structural reconstruction. Because the structure and function of the meniscus are crucial for cartilage protection, the inhibition of meniscus regeneration by ABT‐263 would accelerate cartilage degeneration.

New possibilities for the treatment of meniscus injury emerge from the results of this study. According to our data, inducing the transient accumulation of senescent cells will be a key solution for human meniscus regeneration. For example, treatments could include transplantation of senescent cells and their removal at an appropriate time or the injection of SASP factors derived from senescent cells to activate SFs and induce meniscus healing. The realization of this senescent cell strategy will require further research to unveil the underlying mechanisms.

One major limitation of this study is that we did not identify a specific SASP factor responsible for mediating the activation of SFs. Since we performed bulk RNA‐seq, we could only analyze gene expression changes at the tissue level. Another major limitation was that senescent cells may have different properties in vivo and in vitro (Yagi et al., 2023). These factors will be better understood by conducting RNA‐seq experiments on single cells to assess individual cell states and cell–cell communication in in vivo experiments.

In conclusion, this study investigated the role of cellular senescence in the spontaneous repair of the rat meniscus. Senescent cells transiently accumulated in the regenerating tissue in the early phase after the pMx surgery. Genes related to aging, ECM synthesis, and cell proliferation were highly expressed in the regenerating tissue. SASP factors from senescent SFs promoted the in vitro proliferation and chondrogenesis of normal rat SFs. Selective elimination of senescent SFs with ABT‐263 retarded the spontaneous in vivo meniscus regeneration. Collectively, our results indicated that transient senescent cell accumulation and SASP contributes to the spontaneous repair of the rat meniscus and will provide a novel strategy for meniscus regeneration based on cellular senescence.

## AUTHOR CONTRIBUTIONS

Y. A. provided ideas, performed experiments, and wrote the manuscript. K. E. designed the study, provided ideas, performed experiments, organized the data, and completed the manuscript. I. S. provided ideas and revised the manuscript. All authors read and approved the final manuscript.

## FUNDING INFORMATION

This work was supported by a TMDU priority research areas grant, the Japan Society for the Promotion of Science (JSPS) KAKENHI (22 K16708), and a Takeda Science Foundation grant to Kentaro Endo and by funding from the Japan Agency for Medical Research and Development (JP22gm0010009) to Ichiro Sekiya.

## CONFLICT OF INTEREST STATEMENT

The authors declare no conflicts of interest.

## Supporting information


Data S1.

Data S2.

## Data Availability

The datasets generated and analyzed during the current study are available from the corresponding author on reasonable request. All RNA sequencing datasets were deposited in the National Center for Biotechnology Information's Gene Expression Omnibus with accession number GSE268970.
