# Supplementary material for: Cellular senescence contributes to spontaneous repair of the rat meniscus
Source: Aging Cell. 2024 Oct 22;24(2):e14385. doi: 10.1111/acel.14385 (PMC11822631; doi:10.1111/acel.14385)
Supplement: Supplementary file 1 — Data S1. [file ACEL-24-e14385-s001.pdf]

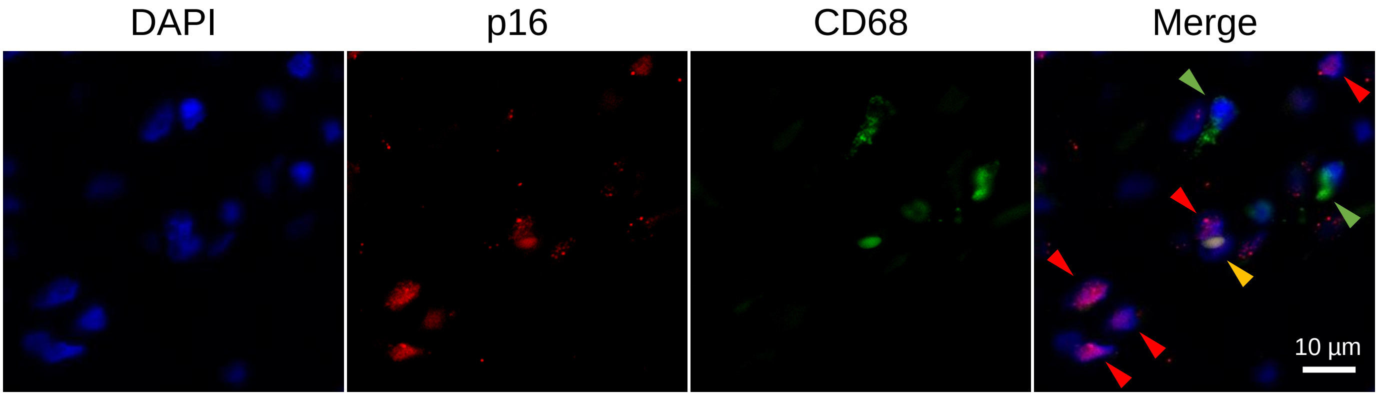

Supplementary Fig. 1 Representative images of p16 and CD68 fluorescence staining of regenerating tissue. Red arrowheads, $p16^{+}CD68^{-}$ cells; green arrowheads, $p16^{-}CD68^{+}$ cells; yellow arrowheads, $p16^{+}CD68^{+}$ cells.

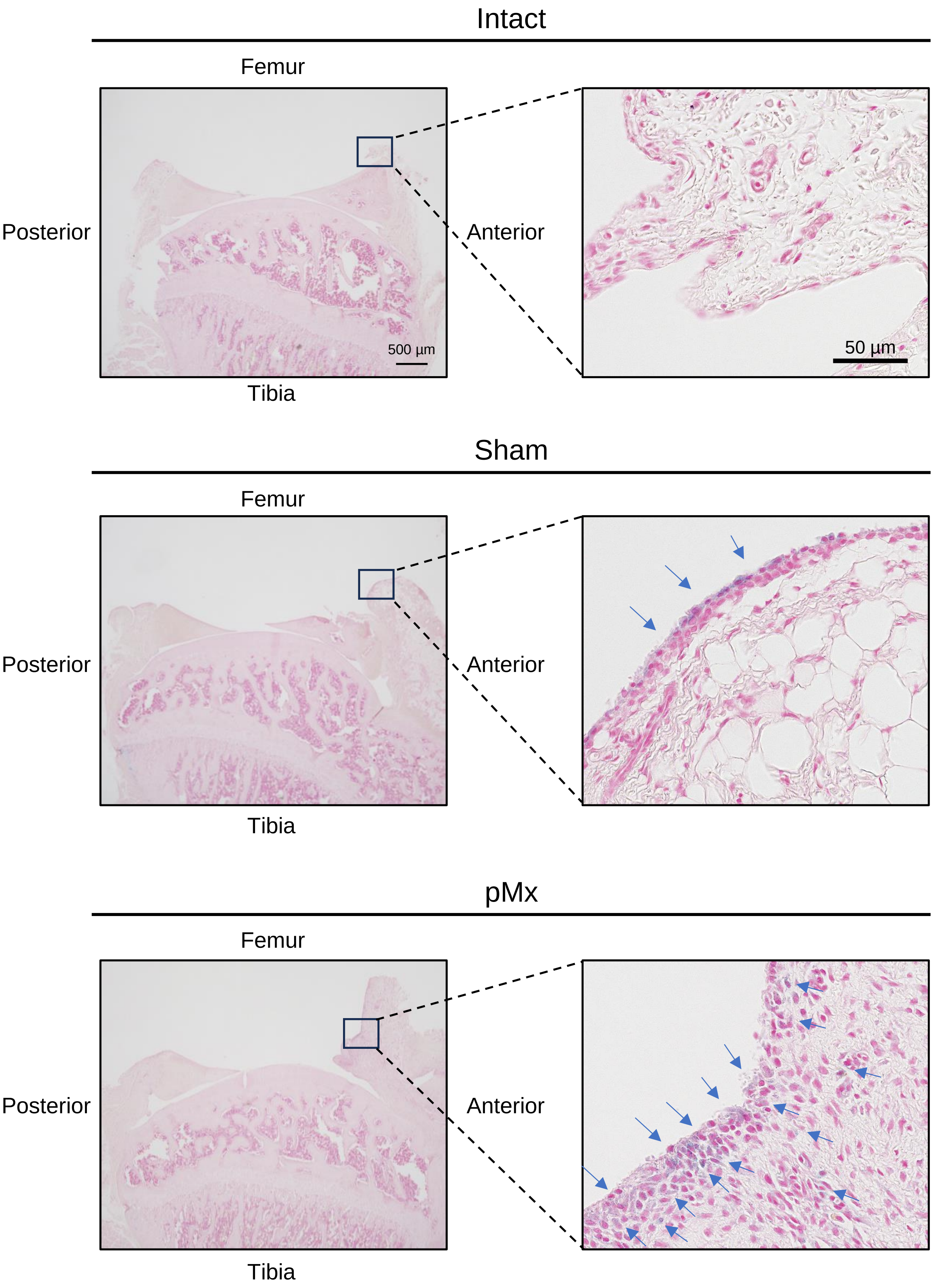

Supplementary Fig. 2 Histological evaluation of knees after SA-β-gal staining. Arrows indicate SA-β gal-positive cells. pMx, partial medial meniscectomy.

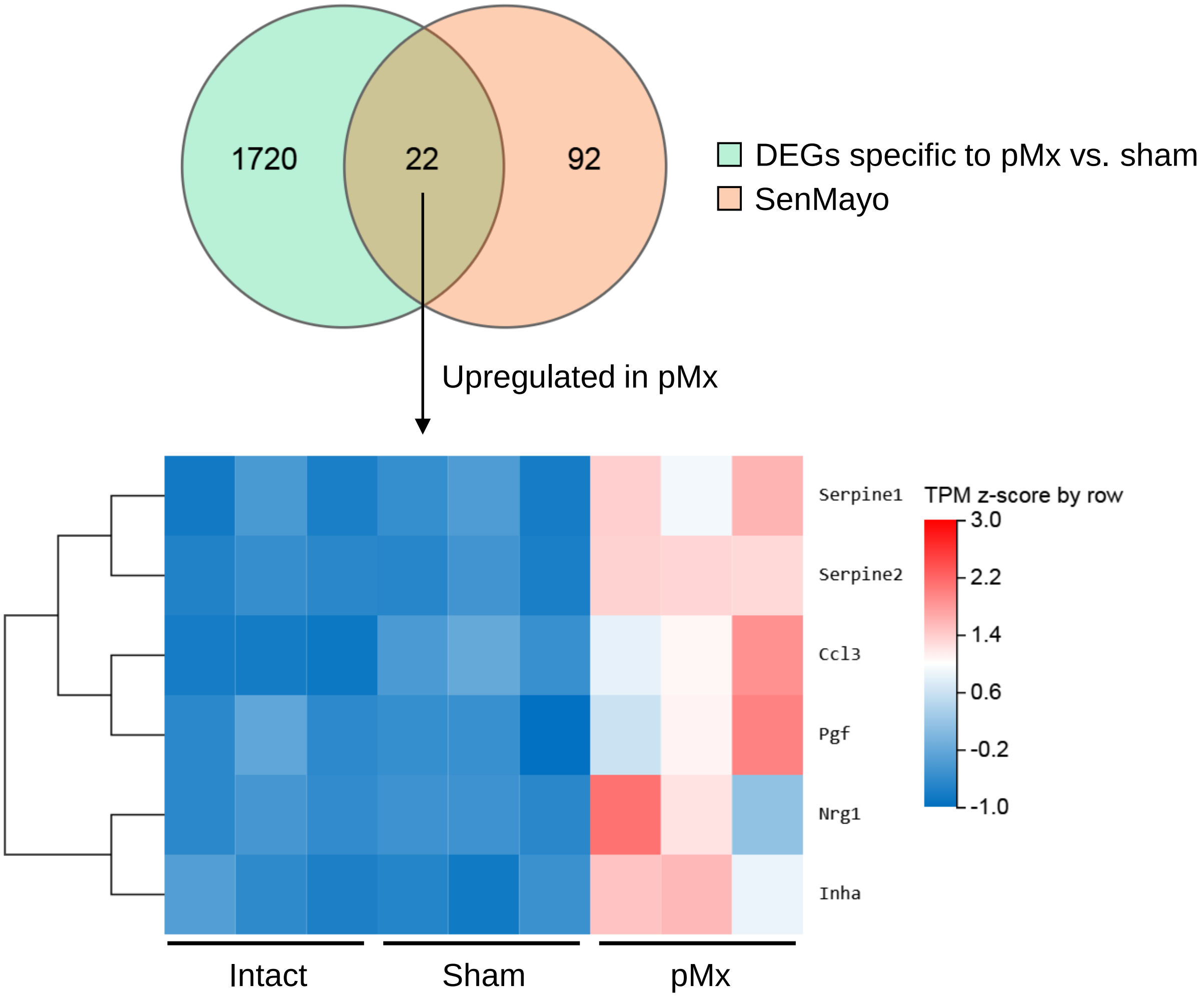

Supplementary Fig. 3. Venn diagram of differentially expressed genes (DEGs) specific to partial medial meniscectomy (pMx) vs. sham and SenMayo. Heatmap shows overlapping genes whose expression was upregulated in pMx.

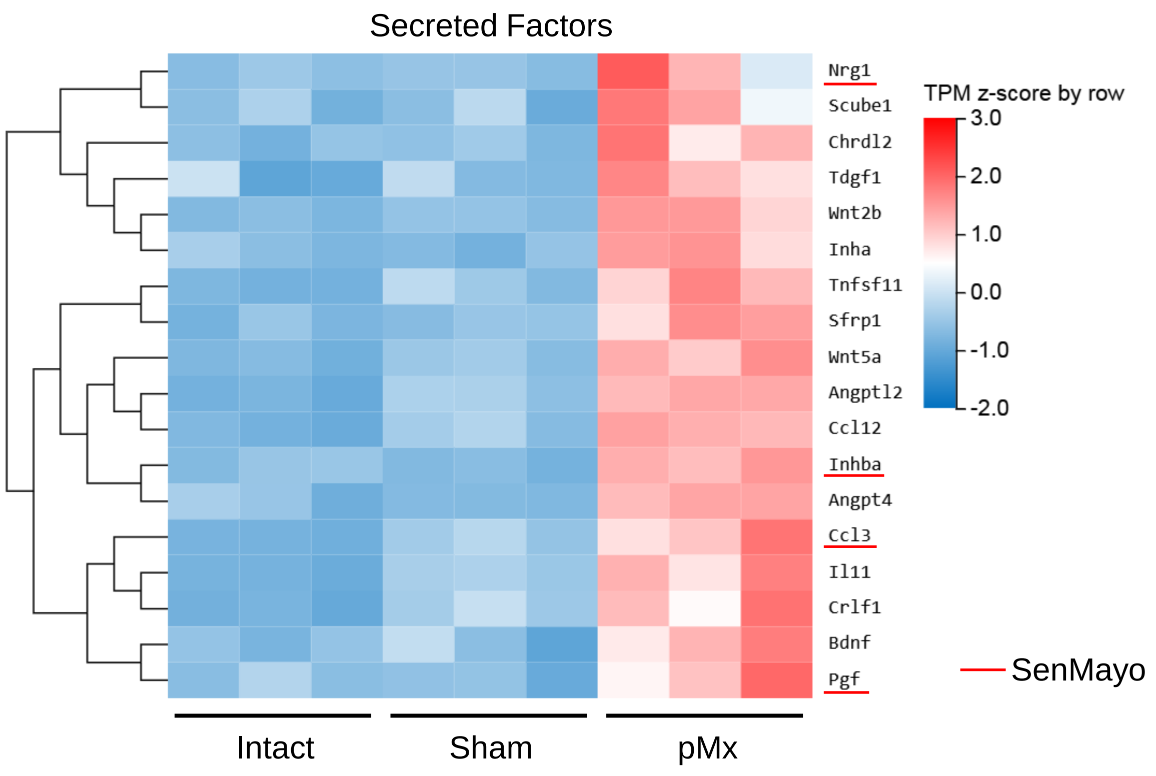

Supplementary Fig. 4 Heatmap of genes coding secreted factors upregulated in the pMx group. Red underlines indicate genes that overlap with SenMayo genes.

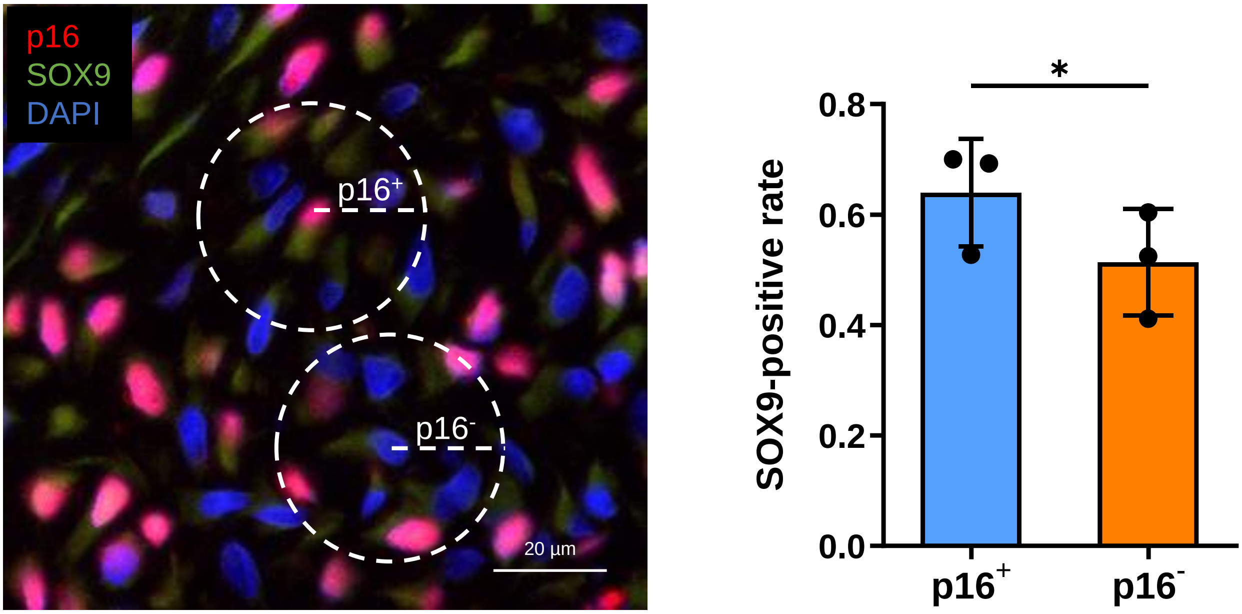

Supplementary Fig. 5. Quantification of the SOX9-positive rate in cells within a 20 µm radius of p16-positive (senescent) and p16-negative (non-senescent) cells. Three knees were evaluated and the p-value was determined using the paired t test.

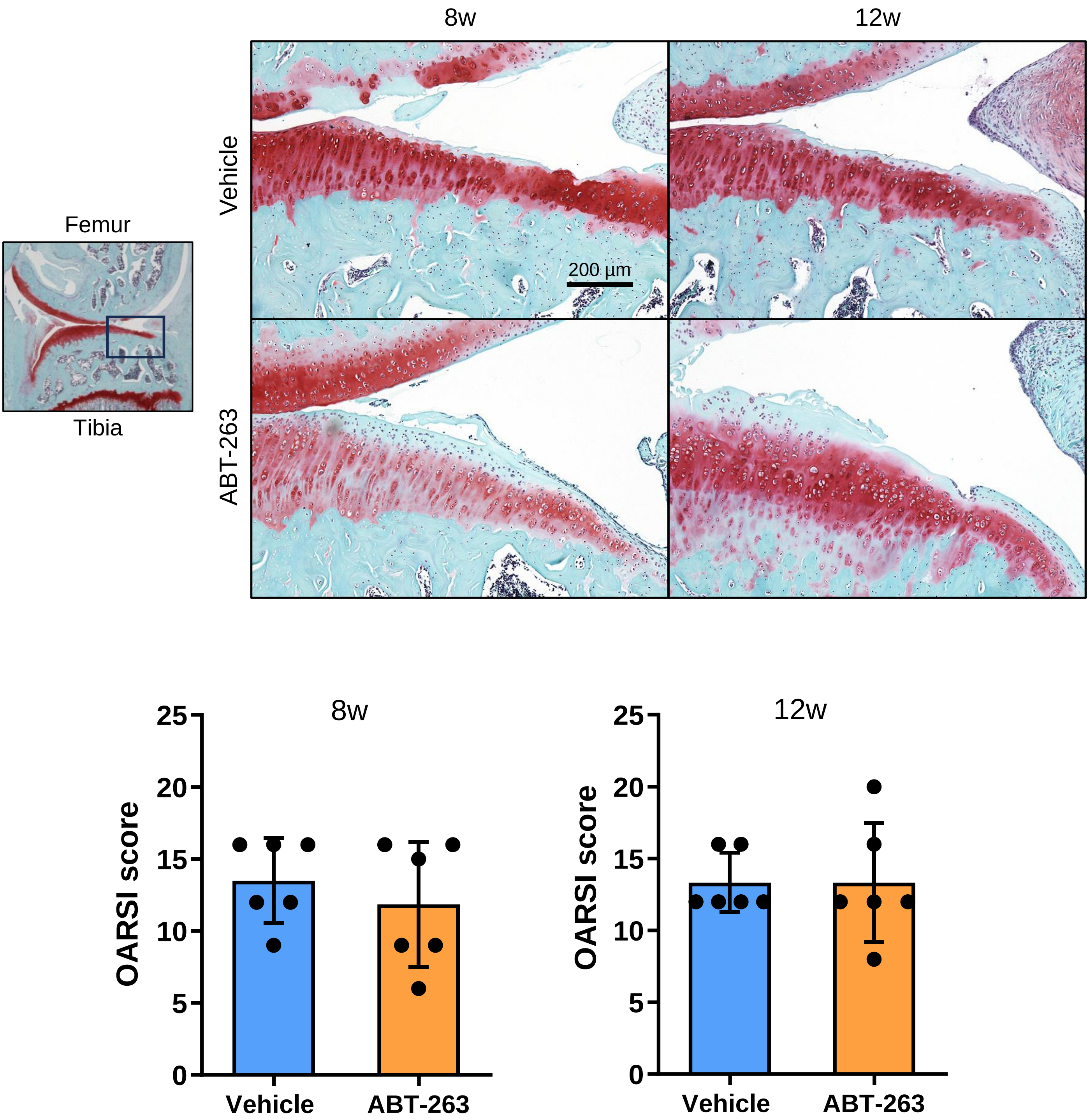

Supplementary Fig. 6 Histological assessment and Osteoarthritis Research Society International (OARSI) scores of the medial tibial plateau cartilage.
