# Supplementary material for: Cellular senescence contributes to spontaneous repair of the rat meniscus
Source: Aging Cell. 2024 Oct 22;24(2):e14385. doi: 10.1111/acel.14385 (PMC11822631; doi:10.1111/acel.14385)
Supplement: Supplementary file 2 — Data S2. [file ACEL-24-e14385-s002.docx]

**Supplementary table 1** Criteria and scores for histological assessment of regenerated menisci (Modified Pauli’s Score, A:3, B:2, C:1, D:0)

| I. Surface including lamellar layer:  I–I. Femoral side:  A Smooth  B Slight fibrillation or slightly undulating  C Moderate fibrillation or markedly undulating  D Severe fibrillation or disruption  I–II. Tibial side  A Smooth  B Slight fibrillation or slightly undulating  C Moderate fibrillation or markedly undulating  D Severe fibrillation or disruption  I–III. Inner border  A Smooth  B Slight fibrillation or slightly undulating  C Moderate fibrillation or markedly undulating  D Severe fibrillation or disruption |
| --- |
| II. Cellularity of meniscal cell  A Normal cell distribution  B Moderately normal cell distribution  C Hypercellularity or hypocellularity  D No meniscal cells |
| III. Collagen fiber organization  A Collagen fibers well organized, no separations or tears  B Collagen fibers moderately organized, slight separations or tears  C Collagen fiber unorganized, moderate separations or tears  D Collagen fiber unorganized, severe separations or tears |
| IV. Matrix staining (safranin-O)  A Well stained like normal meniscus  B Moderately stained  C Slightly stained  D No stain |

**Supplementary table 2** Quality of RNA used for RNA-seq.

| Sample | Concentration  (ng/μL) | Volume  (μL) | Total Mass  (μg) | RIN | 28S/18S |
| --- | --- | --- | --- | --- | --- |
| Intact_1 | 134 | 12 | 1.61 | 6.6 | 1.1 |
| Intact_2 | 80 | 12 | 0.96 | 6.6 | 0.9 |
| Intact_3 | 161 | 12 | 1.93 | 6.7 | 1.3 |
| Sham_1 | 286 | 12 | 3.43 | 7.2 | 1.2 |
| Sham_2 | 554 | 12 | 6.65 | 7 | 1 |
| Sham_3 | 328 | 12 | 3.94 | 7.2 | 1.4 |
| pMx_1 | 851 | 12 | 10.21 | 8.1 | 1.3 |
| pMx_2 | 662 | 12 | 7.94 | 8.7 | 1.5 |
| pMx_3 | 630 | 12 | 7.56 | 8.6 | 1.4 |
